# Pharmacist-led Transitions of Care Intervention to Address Infectious Disease Tests Pending at Discharge (TPAD)

**DOI:** 10.1093/ofid/ofag585

**Published:** 2026-09-12

**Authors:** Elizabeth W Covington, Matthew Shane Loop, Jieun Park, Leborah Lee, Mary Bowman, Sarah Grace Gunter

**Affiliations:** Department of Pharmacy Practice, Auburn University Harrison College of Pharmacy, Auburn, Alabama, USA; Pharmacy Department, East Alabama Health, Opelika, Alabama, USA; Department of Health Outcomes Research and Policy, Auburn University Harrison College of Pharmacy, Auburn, Alabama, USA; Division of Research, Auburn University Harrison College of Pharmacy, Auburn, Alabama, USA; Pharmacy Department, East Alabama Health, Opelika, Alabama, USA; Department of Pharmacy Practice, Auburn University Harrison College of Pharmacy, Auburn, Alabama, USA; Pharmacy Department, East Alabama Health, Opelika, Alabama, USA

**Keywords:** antimicrobial stewardship, continuity of patient care, patient discharge, pharmacists, transitions of care

## Abstract

**Background:**

Infectious disease test results frequently finalize after hospital discharge, and many are clinically actionable. Standardized workflows for reviewing these results remain limited. Although pharmacist-led culture follow-up is well established in emergency departments, evidence evaluating inpatient transitions-of-care models is sparse. This study assessed the impact of a pharmacist-led service on outpatient antimicrobial appropriateness and clinical outcomes.

**Methods:**

We conducted a single-center, retrospective pre–post study at a community hospital comparing patients discharged with pending infectious disease test results before and after service implementation. The intervention included thrice-weekly review of finalized results, clinical assessment, prescriber communication, outpatient prescription changes, and patient counseling. The primary outcome was appropriate outpatient antimicrobial therapy 7 days after discharge. Secondary outcomes included time to appropriate therapy among patients requiring prescription modification and 30-day infection-related outcomes. Multivariable logistic regression adjusted for urine culture source, discharge service, and inpatient infectious diseases consultation. Time-to-therapy analyses used Kaplan–Meier and Cox proportional hazards.

**Results:**

Among 191 patients (93 preintervention; 98 postintervention), appropriate antimicrobial therapy at 7 days increased from 69% to 92%. Adjusted analyses showed a 21.3% higher predicted probability of appropriate therapy (95% CI, 10.6–32.0). Among patients requiring prescription changes (n = 63), median time to appropriate therapy improved from 7 to 4 days, with a 5-fold higher rate of achieving appropriate therapy (hazard ratio, 5.09; 95% CI, 2.13–12.18). Thirty-day outcomes were similar between groups.

**Conclusions:**

A pharmacist-led service improved antimicrobial appropriateness and accelerated transitions to appropriate therapy, supporting pharmacist-driven follow-up as an effective stewardship strategy.

## Teaser

A pharmacist-led service reviewing infectious disease test results pending at discharge improved outpatient antimicrobial appropriateness and shortened time to necessary therapy changes. This structured follow-up addressed communication gaps in care transitions, supporting safer postdischarge antimicrobial management.

Over 40% of hospitalized patients will have tests pending at discharge (TPAD) [1–3]. Infectious disease test results, such as microbiological culture data, are one of the most common subsets of TPAD. These results may indicate that a patient needs a change in management, such as a different antimicrobial regimen. Failure to act on these results after hospital discharge may contribute to adverse outcomes, including worsening infection, hospital readmission, and adverse drug events. Poor follow-up of TPAD has been recognized as an important patient safety issue [3, 4].

Ensuring timely review and follow-up of TPAD remains challenging. During transitions of care, responsibility for reviewing pending results may be unclear, and communication failures between inpatient and outpatient providers are common. In a survey of outpatient providers receiving discharge summaries, more than half of reported preventable errors were related to communication failures during transitions of care, including missed TPAD results [5]. Follow-up of TPAD is hindered by unclear ownership of results, poor communication between inpatient and outpatient clinicians, and limited recognition of the urgency or clinical importance of pending abnormalities. These challenges are further compounded by hospitalist-based care, in which the ordering clinician may no longer be on service when results become available. Additional factors complicating TPAD follow-up include limited direct communication between hospital and primary care physicians, incomplete discharge summaries, and reliance on passive systems—such as discharge-summary documentation, electronic notifications, or patient portal access—without active clinical follow-up [1, 6].

Evidence to inform optimal TPAD follow-up workflows remains limited. Existing interventions have largely focused on discharge documentation templates and electronic notification systems [1, 2, 7, 8]. Although these interventions have improved documentation and awareness of TPAD, a substantial proportion of pending results remained undocumented or unacknowledged even after intervention [2]. Importantly, improving documentation does not necessarily ensure timely clinical review or action on actionable results. In addition, literature evaluating the effect of TPAD-focused interventions on patient outcomes remains sparse.

Pharmacists may be particularly well suited to support TPAD follow-up involving infectious disease test results because interpretation of these results often requires assessment of antimicrobial selection, spectrum of activity, dose, duration, safety, and need for de-escalation or escalation. These responsibilities align closely with established pharmacist roles in antimicrobial stewardship [9]. Pharmacist involvement in postdischarge culture follow-up is well established in the emergency department (ED) setting, where these services have demonstrated positive effects on workflow and patient outcomes [10]. However, despite the frequency and clinical importance of TPAD in acutely ill hospitalized patients, data describing pharmacist-led TPAD follow-up remain extremely limited. Jones et al conducted a comparative pilot study demonstrating that pharmacist review could identify actionable results requiring antimicrobial modification [6]. Van Abel et al subsequently described a large retrospective pharmacist-led postdischarge microbiology review service without a comparator group [11]. Comparative effectiveness data therefore remain sparse.

Given the frequency and clinical importance of infectious disease TPAD, the workflow challenges surrounding postdischarge result follow-up, and the limited evidence supporting structured inpatient follow-up processes, this study aimed to evaluate the impact of a pharmacist-led service for infectious disease TPAD.

## METHODS

### Study Design and Site

This was a single center, retrospective pre–post study at East Alabama Medical Center (EAMC), a 316-bed community not-for-profit hospital in Opelika, Alabama, from August 2022 through October 2024. EAMC has maintained an antimicrobial stewardship program since 2010 and is an Infectious Diseases Society of America Center of Excellence. The antimicrobial stewardship team includes a clinical pharmacist serving as antimicrobial stewardship coordinator and a physician co-lead. In addition, a clinical pharmacy faculty member practices with the antimicrobial stewardship team ∼12 hours weekly. ED pharmacists also perform culture follow-up services in the ED, independent of the inpatient TPAD intervention, as described in a previous publication [12]. During the preintervention period, no standardized workflow or designated pharmacist was responsible for reviewing infectious disease tests that finalized after inpatient discharge. Follow-up depended on usual clinical workflows, without a formally designated individual or service responsible for ensuring review and action. Pharmacists could become involved if a result was identified through routine clinical activities (such as review of blood cultures) or if assistance was requested, but systematic pharmacist identification and review of infectious disease TPAD did not occur.

### Intervention

The TPAD service is described below using the Template for Intervention Description and Replication (TIDieR) framework [13]. A pharmacist-led TPAD service intervention was implemented in October 2023. The purpose of the intervention was to identify patients with infectious diseases tests that became available after hospital discharge, assess the clinical relevance of those results, and facilitate postdischarge antimicrobial changes when indicated. Although the TPAD intervention was not explicitly designed using a formal implementation framework, its development and execution were conceptually consistent with the Integrated Promoting Action on Research Implementation in Health Services framework, with pharmacist facilitation serving as the primary implementation strategy; this alignment is summarized in Supplementary Table 1.

The intervention was provided primarily by a clinical pharmacist who has completed postgraduate residency training and holds board certification in infectious diseases pharmacy. The pharmacist reviewed TPAD results 3 mornings per week (Monday, Tuesday, and Thursday), in addition to other clinical antimicrobial stewardship tasks. If positive blood cultures resulted after discharge on days outside this schedule, a full-time antimicrobial stewardship pharmacist intervened. Other infectious diseases test results that became available outside scheduled review days were addressed on the next scheduled TPAD service day. Fourth-year pharmacy students with active intern licenses also participated under pharmacist supervision for ∼25 weeks annually by assisting with chart review, facilitating new prescriptions, and counseling patients.

The TPAD workflow included 5 components: (1) identification of patients with infectious disease TPAD, (2) pharmacist assessment of TPAD results in clinical context, (3) communication of recommendations to the discharging or responsible clinician, (4) facilitation of outpatient prescription changes when needed, and (5) patient counseling regarding therapy changes.

Theradoc^TM^ clinical surveillance software (Salt Lake City, Utah) was utilized to identify patients with postdischarge infectious disease results. Specifically, Theradoc^TM^ Therapeutic Antimicrobial Monitoring (TAM) alerts with patient location at specimen collection “inpatient” and patient location at specimen result “not inpatient” were reviewed. Additionally, the pharmacist reviewed a report within Theradoc^TM^ of positive microbiology results within the last 7 days, filtered by discharged patients, to capture any results that did not trigger a TAM alert. Examples of results that may not generate a TAM alert but could be identified using this secondary review method include positive *Helicobacter pylori* results and finalized anaerobic culture growth after discharge.

Postdischarge antimicrobial recommendations were communicated to the responsible prescriber for approval; pharmacists did not independently modify therapy under a collaborative practice agreement. Communication with providers occurred via electronic secure messaging or telephone. In most cases, the pharmacist would first attempt to contact the discharging provider. If the provider was no longer on service, the pharmacist would contact a nurse coordinator to discuss the most appropriate provider to contact. Telephone was used to contact patients and communicate with outpatient pharmacies, if needed. The pharmacist called the patient to provide laboratory results and education on prescription changes. If unable to contact the patient or leave a voicemail, the pharmacist would call the emergency contact and request the patient call back. Attempts to call the patient would be repeated 2–3 times. Intervention activities were documented in the electronic health record (EHR) and TheraDoc^TM^.

The intervention was tailored according to whether a postdischarge antimicrobial change was clinically warranted. If the discharge antimicrobial was considered appropriate, no intervention was made. Appropriate antimicrobial therapy was defined as an antimicrobial with in vitro activity against the identified organism(s) that reaches the site of infection, prescribed at an appropriate dose and frequency according to EAMC antimicrobial dosing criteria or UpToDate® Lexidrug™ (if not listed in EAMC dosing criteria), and with a duration of therapy that was not shorter than evidence-based guideline recommendations. Therapy was not classified as inappropriate solely because the regimen was broader in spectrum or longer in duration than necessary if it remained clinically acceptable. The stewardship committee decided a priori not to intervene solely to de-escalate or shorten therapy postdischarge in situations where the existing regimen remained clinically acceptable, in part to avoid unnecessary burden or cost to patients. Similarly, certain postdischarge results were predefined as not requiring clinician notification. For example, positive urine cultures in patients without urinary symptoms or systemic signs of infection were considered asymptomatic bacteriuria and were not reported to clinicians to avoid promoting unnecessary treatment [14].

### Patients/Participants

Patients at EAMC with infectious disease TPAD were compared before and after implementation of the pharmacist-led TPAD service. Eligible patients were identified through retrospective reports generated from the EHR (Cerner^TM^) and Theradoc^TM^. Patients were eligible if they (1) had an inpatient or observation encounter at EAMC between August 2022 and October 2024, (2) underwent microbiologic, polymerase chain reaction (PCR), serologic, or other infectious diseases testing at EAMC, (3) were discharged alive and not to hospice or comfort care, and (4) had at least 1 infectious diseases test that resulted after discharge. Study exclusion criteria were: age ≤18 years, prisoner status, transfer to another acute care hospital at discharge, death before postdischarge infectious diseases results became available, blood culture contamination, *Candida* species in sputum, *Pseudomonas* species in stool, vaginal group B streptococcal swabs, and urine cultures with ≥3 species. The latter microbiologic exclusions were added by IRB addendum as examples of TPAD not expected to require intervention.

### Outcomes

The primary effectiveness outcome was appropriate antimicrobial therapy at 7 days postdischarge, regardless of whether a prescription change was needed. This outcome was intended to identify clinically meaningful under-treatment, untreated infection, drug–organism mismatch, or dosing and duration deficiencies; it did not require the narrowest possible spectrum or shortest acceptable duration. The 7-day time point was selected to allow pending infectious disease results to finalize and indicated treatment changes to be implemented while maintaining focus on the early postdischarge period. The 7-day window also accommodated the thrice-weekly TPAD review schedule. To reduce assessment bias, antimicrobial appropriateness was adjudicated by an infectious disease trained pharmacist who was not involved in delivering the TPAD intervention. The adjudicator was not blinded to study period because group assignment was available in the study dataset and could also be inferred from encounter dates. When the adjudicator's assessment differed from the initial assessment performed during data collection, the adjudicator documented the rationale for the final determination. In cases requiring clarification of the outcome definition or usual institutional practice, an additional investigator was consulted; these consultations were used sparingly to minimize potential bias. Formal interrater agreement was not assessed because the initial reviewer and adjudicator differed substantially in training and clinical expertise and were not intended to function as equivalent independent raters.

The outpatient antimicrobial regimen at 7 days after discharge was determined through review of available prescription claims, medication orders, physician progress notes updated to document action on the TPAD result or resulting prescription changes, and/or a structured form within the EHR used to document TPAD culture follow-up actions. The most recently documented antimicrobial regimen prescribed on or before postdischarge day 7 was used to adjudicate therapy appropriateness.

Secondary effectiveness outcomes were (1) appropriate antimicrobial therapy at 7 days postdischarge among patients who required a postdischarge prescription change and (2) time to appropriate therapy within 7 days after the postdischarge infectious disease test result became available among patients who required a postdischarge prescription change. Clinical outcomes included a composite treatment failure outcome defined as infection-related readmission or ED visit or microbiological failure (defined as growth of the same organism from the same culture type) within 30 days of discharge, as well as each component outcome individually.

Implementation and process measures included the proportion of patients with TPAD reviewed by a pharmacist or provider, the proportion requiring antimicrobial intervention, reason for intervention (drug/bug mismatch, dose/frequency, duration, and other), number and types of pharmacist interventions performed, documentation of result acknowledgement or action in Cerner, and time to documentation.

### Statistical Analyses

The primary exposure was study period (preintervention vs postintervention). For the primary outcome of appropriate outpatient antimicrobial therapy at 7 days postdischarge, logistic regression was used to estimate the association between the study period and appropriate prescribing with unadjusted and adjusted models. The adjusted model included clinically relevant covariates: urine test source, hospitalist/internal medicine discharge service, and inpatient infectious diseases consultation. In addition to odds ratios (ORs) and 95% confidence intervals (CIs), adjusted predicted probabilities of appropriate antimicrobial therapy and absolute differences in predicted probability of appropriate antimicrobial therapy were calculated. A prespecified secondary logistic regression analysis was performed among the subgroup of patients whose outpatient antimicrobial prescription required postdischarge modification.

Among patients requiring a postdischarge prescription change, time to appropriate therapy was analyzed as a time-to-event outcome, with achievement of appropriate therapy as the event of interest and administrative censoring at 7 days. Kaplan–Meier methods were used to describe time to appropriate therapy by study period, and a Cox proportional hazards model was used to estimate the hazard ratio (HR) for the study period (postintervention vs preintervention). HRs with 95% CIs were reported, with HR >1 indicating a faster rate of achieving appropriate therapy in the postintervention period compared to the preintervention period at any given time point. Each 30-day clinical outcome was analyzed using unadjusted logistic regression among all patients. Results are reported as OR with 95% CIs and absolute difference percentage with 95% CIs.

To examine whether the need for postdischarge antimicrobial modification differed by test source, microbiology source was categorized as PCR, urine, tissue, or other. Because expected cell counts were small in some categories, Fisher's exact test was used to compare the proportion of patients requiring prescription modification across groups. All tests were 2-sided, and a *P* value <.05 was considered statistically significant.

An a priori sample size calculation was performed for the primary outcome of appropriate antimicrobial therapy at 7 days postdischarge. Based on prior literature, ∼36% of infectious diseases tests resulting after discharge were expected to require intervention, physician follow-up in the absence of a structured TPAD intervention was estimated at 30%–40%, and pharmacist involvement was expected to increase action on actionable results to ∼80% [4, 6–8]. Under these assumptions, 96 patients per group were required to provide 80% power with a 2-sided α of .05 to detect an OR of 3.7, corresponding to an estimated increase in the probability of appropriate antimicrobial therapy from 78% in the preintervention group to 93% in the postintervention group. Sample size calculations were performed using PASS 2024 (version 24.0.2).

This study was approved by the appropriate institutional review board(s) with a waiver of informed consent [East Alabama Health protocol 24-27-E; Auburn University protocol STUDY00000310].

## RESULTS

**Figure 1.**
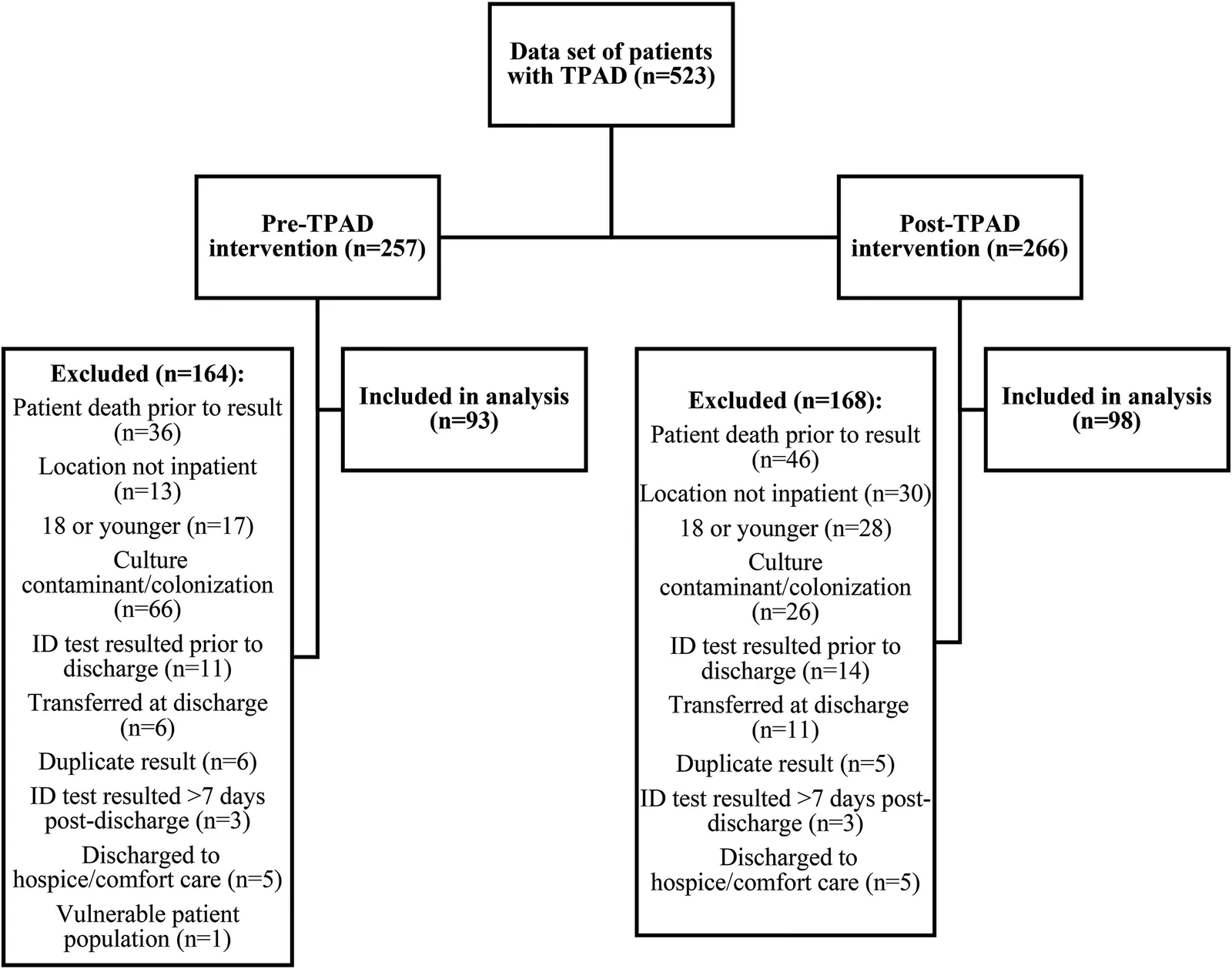
Inclusion Flowchart.

A total of 523 patients with infectious diseases TPAD were screened, of whom 191 were included in the final analysis: 93 in the preintervention period and 98 in the postintervention period (Figure 1). Exclusions were primarily due to death before result availability, testing from a non-inpatient location, age ≤18 years, culture contamination or colonization, and infectious disease test result prior to discharge.

Baseline characteristics were broadly similar between the preintervention and postintervention groups (Table 1). The sample was predominantly middle-aged, female, white, and insured. Hospitalist/internal medicine was the most common discharging service. Urinary tract infection and skin and soft-tissue infection were the most common infection types.

**Table 1.** Patient Characteristics

Variable	Overall (N = 191)	Preintervention (N = 93)	Postintervention (N = 98)	*P* Value^a^
Age (years)	57 (37, 70)	56 (38, 71)	58 (37, 69)	>.9
Female	112 (58.6)	55 (59.1)	57 (58.2)	.9
Race				>.9
White	119 (62.3)	59 (63.4)	60 (61.2)	…
African American	60 (31.4)	28 (30.1)	32 (32.7)	…
Other	12 (6.3)	6 (6.5)	6 (6.1)	…
Patient has insurance	170 (89.0)	84 (90.3)	86 (87.8)	.6
Infectious disease consulted during inpatient stay	42 (22.0)	19 (20.4)	23 (23.5)	.6
Discharge service				.10
Hospitalist/internal medicine	85 (44.5)	46 (49.5)	39 (39.8)	…
Surgery	21 (11.0)	12 (12.9)	9 (9.2)	…
Residency team (IMRT)	17 (8.9)	4 (4.3)	13 (13.3)	…
Other	68 (35.6)	31 (33.3)	37 (37.8)	…
Length of stay in days	3.0 (2.0, 6.0)	3.0 (2.0, 6.0)	3.0 (1.0, 6.0)	.3
Outpatient antimicrobial prescribed at discharge	149 (78.0)	71 (76.3)	78 (79.6)	.6
Infection type				.2
UTI	65 (34.0)	29 (31.2)	36 (36.7)	…
SSTI	51 (26.7)	23 (24.7)	28 (28.6)	…
Bone/joint	23 (12.0)	13 (14.0)	10 (10.2)	…
Intra-abdominal	13 (6.8)	6 (6.5)	7 (7.1)	…
Pneumonia	12 (6.3)	3 (3.2)	9 (9.2)	…
STI	5 (2.6)	3 (3.2)	2 (2.0)	…
Other	17 (8.9)	12 (12.9)	5 (5.1)	…
None	5 (2.6)	4 (4.3)	1 (1.0)	…
Type of laboratory test				.6
Tissue culture	70 (36.6)	32 (34.4)	38 (38.8)	…
Urine culture	65 (34.0)	31 (33.3)	34 (34.7)	…
Respiratory culture	23 (12.0)	10 (10.8)	13 (13.3)	…
Abdominal fluid culture	16 (8.4)	9 (9.7)	7 (7.1)	…
PCR-based test	8 (4.2)	5 (5.4)	3 (3.1)	…
Blood culture	5 (2.6)	2 (2.2)	3 (3.1)	…
Stool culture	3 (1.6)	3 (3.2)	0 (0.0)	…
Other	1 (0.5)	1 (1.1)	0 (0.0)	…
Organism category				.005
Gram-negative	82 (42.9)	37 (39.8)	45 (45.9)	…
Gram-positive	46 (24.1)	29 (31.2)	17 (17.3)	…
Polymicrobial	46 (24.1)	18 (19.4)	28 (28.6)	…
Fungal	8 (4.2)	1 (1.1)	7 (7.1)	…
Anaerobe	7 (3.7)	6 (6.5)	1 (1.0)	…
Other	2 (1.0)	2 (2.2)	0 (0.0)	…

Continuous variables are reported as Median (Q1, Q3); Categorical variables are reported as n (%).

The tissue category included wound, abscess, operative, bone, and other deep tissue specimens.

Abbreviations: IQR, interquartile range; no., number; PCR, polymerase chain reaction; SD, standard deviation; SSTI, skin and soft-tissue infection; STI, sexually transmitted infection; TPAD, tests pending at discharge; UTI, urinary tract infection.

^a^Fisher's exact test was used for infection type, type of laboratory test, and organism category. For the remaining categorical variables, Pearson's χ^2^ test was used. Wilcoxon rank-sum test was used for continuous variables.

In the primary analysis of all patients with TPAD, the postintervention period was associated with more appropriate outpatient antimicrobial therapy at 7 days postdischarge in both unadjusted and adjusted logistic regression models (Table 2). The proportion of patients with appropriate therapy increased from 68.8% before the intervention to 91.8% after the intervention. In the adjusted model, the postintervention period was associated with a 21.3-percentage-point higher predicted probability of appropriate therapy (95% CI, 10.6–32.0), after accounting for urine test source, hospitalist/internal medicine discharge service, and inpatient infectious diseases consultation.

**Table 2.** Unadjusted and Adjusted Differences in Appropriate Outpatient Antimicrobial

Patient Group	Analysis Type	Overall	Preintervention	Postintervention	Absolute Difference (95% CI)^a^	OR (95% CI)^b^
All patients (n = 191)	Unadjusted^c^	80.6 (154/191)	68.8 (64/93)	91.8 (90/98)	+ 23.0 (12.2, 33.9)	5.10 (2.28, 12.62)
	Adjusted^d^	…	71.1	92.4	+ 21.3 (10.6, 32.0)	4.94 (2.18, 12.40)
Patients requiring prescription change postdischarge (n = 63)	Unadjusted^c^	41.3 (26/63)	19.4 (7/36)	70.4 (19/27)	+ 50.9 (29.4, 72.5)	9.84 (3.21, 33.85)
	Adjusted^d^	…	18.0	71.8	+ 53.8 (31.5, 76.0)	11.57 (3.53, 45.09)

^a^Absolute difference from logistic regression predicted probabilities (postintervention minus preintervention).

^b^Odds ratio from logistic regression; reference = preintervention.

^c^Reported as n (%).

^d^Reported as %.

In the prespecified secondary analysis restricted to the 63 patients who required a postdischarge antimicrobial prescription change, the postintervention period was likewise associated with improved appropriate outpatient antimicrobial therapy at 7 days postdischarge (Table 2). The proportion of patients with appropriate therapy increased from 19.4% in the preintervention period to 70.4% in the postintervention period. In both unadjusted and adjusted logistic regression models, the postintervention period remained associated with higher odds of appropriate therapy.

**Figure 2.**
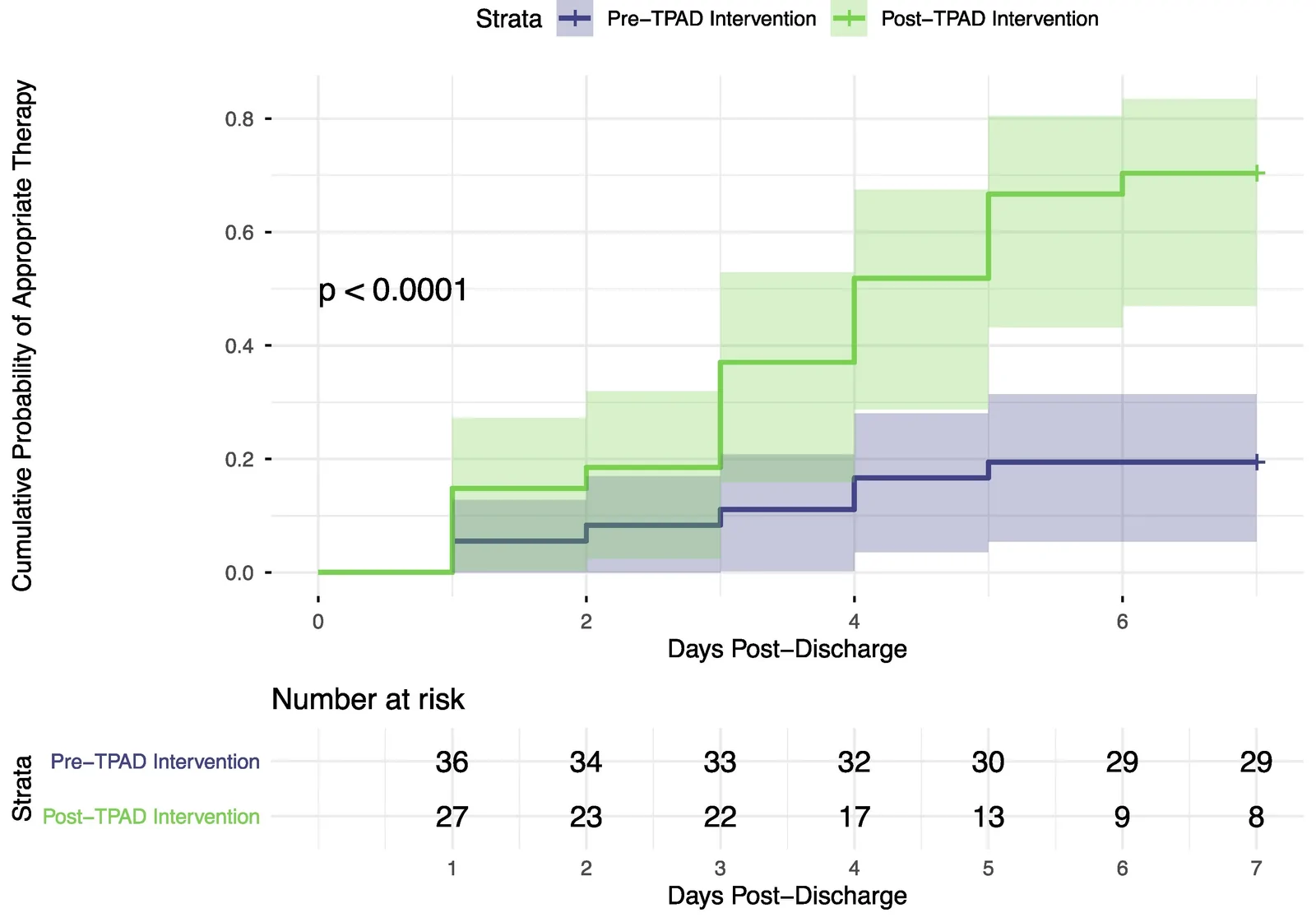
In this analysis, the “event” is defined as achieving appropriate therapy. A hazard ratio >1 indicates a faster transition to appropriate prescription. Abbreviations: CI, confidence interval; HR, hazard ratio.

Among patients requiring a prescription change (n = 63), the median time to appropriate therapy was shorter in the postintervention group compared to the preintervention group (4 days vs 7 days). In the Kaplan–Meier analysis, 50% of patients in the postintervention group achieved appropriate therapy by day 4, whereas the preintervention group failed to reach a 50% cumulative success rate within 7 days (Figure 2). Consistent with this finding, the Cox proportional hazards model showed that the postintervention period was associated with a significantly faster rate of achieving appropriate therapy (HR, 5.09; 95% CI, 2.13–12.18).

The proportion of patients requiring a postdischarge antimicrobial prescription change varied by microbiology test source (Fisher's exact test, *P* < .001). Observed rates of needing a postdischarge antimicrobial prescription change were: PCR (8/8, 100%); urine (18/65, 27.7%); tissue (21/70, 30.0%); and all other sources (16/48, 33.3%). However, no separate hypothesis test was performed to compare PCR results against the other source categories.

The impact of the TPAD intervention on 30-day clinical outcomes and healthcare utilization is presented in Table 3. Thirty-day infection-related events were infrequent in both groups, and no statistically significant differences were observed in infection-related readmission, ED visits, microbiological failure, or the composite treatment failure outcome.

**Table 3.** Thirty-Day Clinical Outcomes

Outcome	Overall (n = 191)^a^	Preintervention (n = 93)^a^	Postintervention (n = 98)^a^	Absolute Difference (95% CI)^b^	*P* Value^c^	OR (95% CI)^d^
Infection-related readmission	10 (5.2)	6 (6.5)	4 (4.1)	−2.4 (−8.7, 4.0)	.464	0.62 (0.15, 2.23)
ED visit	3 (1.6)	2 (2.2)	1 (1.0)	−1.1 (−4.7, 2.4)	.533	0.47 (0.02, 4.98)
Microbiological failure	4 (2.1)	1 (1.1)	3 (3.1)	+2.0 (−2.0, 6.0)	.331	2.91 (0.36, 59.32)
Treatment failure^e^	12 (6.3)	6 (6.5)	6 (6.1)	−0.3 (−7.2, 6.6)	.925	0.95 (0.29, 3.13)

^a^Reported as n (%).

^b^Absolute difference from logistic regression predicted probabilities (postintervention minus preintervention).

^c^ *P* value for the absolute difference (marginal effect from logistic regression).

^d^Odds ratio from logistic regression; reference = preintervention.

^e^Treatment failure was defined as infection-related readmission, emergency department visit, or microbiologic failure within 30 days postdischarge.

Process measures are summarized in Table 4. Pharmacist review of TPAD increased markedly after implementation of the service, from 3.2% of cases in the preintervention period to 87.8% in the postintervention period. The 12 postintervention cases without pharmacist review were not captured through the TheraDoc alert or secondary report during routine service delivery and were subsequently identified through the broader retrospective research report. Potential reasons included changes in hospital unit names that required manual updating of alert parameters and gaps following pharmacist absence when the report look-back period was not extended sufficiently; however, the specific reason could not be determined for every case. Across both periods, the most common reasons for intervention were drug-bug mismatch and untreated indication. In the postintervention period, pharmacist intervention types included chart review, provider notification, patient counseling, and facilitation of outpatient prescription changes, with a corresponding increase in total TPAD-related pharmacist interventions and more timely documentation in the EHR. Among 21 postintervention pharmacist antimicrobial recommendations, 17 (81.0%) were accepted, 1 (4.8%) was not accepted, and provider response could not be determined for 3 (14.3%). Among recommendations with a documented provider response, 17 of 18 (94.4%) were accepted.

**Table 4.** Implementation and Process Measures for the Pharmacist-Led TPAD Service

Process Measures	Overall (N = 191)	Preintervention (N = 93)	Postintervention (N = 98)
Patients with TPAD reviewed by pharmacist	89 (46.6)	3 (3.2)	86 (87.8)
Patients requiring antimicrobial intervention	63 (33.0)	36 (38.7)	27 (27.6)
Reason antimicrobial intervention needed among patients requiring intervention			
Dose/frequency issue	5 (7.9)	1 (2.8)	4 (14.8)
Drug/bug mismatch	35 (55.6)	21 (58.3)	14 (51.9)
Duration issue	1 (1.6)	0 (0.0)	1 (3.7)
Untreated indication	21 (33.3)	13 (36.1)	8 (29.6)
Other	1 (1.6)	1 (2.8)	0 (0.0)
Pharmacist intervention category			
Review only	61 (31.9)	2 (2.2)	59 (60.2)
Antimicrobial recommendation	22 (11.5)	1 (1.1)	21 (21.4)
Patient counseling	10 (5.2)	0 (0.0)	10 (10.2)
Prescription transmitted to outpatient pharmacy to facilitate medication access	11 (5.8)	0 (0.0)	11 (11.2)
Provider notified of lab result without recommendation	20 (10.5)	0 (0.0)	20 (20.4)
Lab clarification	4 (2.1)	0 (0.0)	4 (4.1)
Total number of TPAD-related pharmacist intervention	128.00	3.00	125.00
Patient chart with documentation of TPAD result acknowledgment or action in Cerner	65 (34.0)	20 (21.5)	45 (45.9)
Hours from result to documentation of result acknowledgement (censored at 168 h/7 d)	168 (74, 168)	168 (168, 168)	168 (32, 168)
Time to appropriate therapy in days	7.00 (4.00, 7.00)	7.00 (7.00, 7.00)	4.00 (3.00, 7.00)

Continuous variables reported as Median (Q1, Q3); Categorical as n (%).

Pharmacist intervention category: Percentages are calculated based on the total number of patients in each group; patients may receive multiple interventions.

## DISCUSSION

A pharmacist-led intervention to address infectious diseases TPAD was associated with improved outpatient antimicrobial appropriateness at 7 days postdischarge and a faster transition to appropriate therapy among patients requiring postdischarge prescription modification. In the overall cohort, the postintervention period was associated with a substantially higher predicted probability of appropriate outpatient antimicrobial therapy at 7 days, with an even more pronounced effect among patients requiring a prescription change. Importantly, the TPAD service was operationalized with limited dedicated pharmacist time (thrice weekly) and student support during part of the year, yet still achieved review of nearly 90% of TPAD cases in the postintervention period, supporting the feasibility and sustainability of this approach in a community hospital setting.

Despite the substantial improvement in antimicrobial appropriateness and timeliness, we did not observe differences in 30-day infection-related outcomes. Among patients requiring prescription modification, the majority of pharmacotherapy issues involved drug–organism mismatch or an untreated indication, and most occurred among patients with nonurinary test sources downstream infection-related events were infrequent, and the study was powered for antimicrobial appropriateness rather than clinical outcomes. Accordingly, the absence of differences in 30-day outcomes should not be interpreted as evidence that these prescribing deficiencies were clinically inconsequential. Larger, multisite studies will be needed to determine whether improvements in antimicrobial appropriateness and timeliness translate into reductions in clinical outcomes such as infection-related readmission or microbiologic failure.

Our findings add to a limited but growing body of literature describing stewardship-focused follow-up of postdischarge infectious diseases results. Prior work by El-Kareh et al reported that only a small proportion of postdischarge culture results were clinically important and untreated at discharge, with an even smaller proportion necessitating antimicrobial modification [8]. In our cohort, the proportion requiring postdischarge prescription change was considerably higher at 33.0% (Table 4), similar to the findings of Van Abel et al [11]. This difference may reflect variation in study populations, test types included, local prescribing practices, and case ascertainment strategies. El-Kareh et al also identified urine cultures as a predictor of actionable postdischarge results, whereas in our study urine cultures had the lowest proportion requiring prescription modification across microbiology source groups [15]. This contrast suggests that the types of actionable TPAD may vary substantially by institution and reinforces the importance of local data when designing follow-up services.

Our results also reinforce the encouraging results of prior intervention studies focused on postdischarge results follow-up. In a pilot study by Jones et al, antimicrobial stewardship pharmacist involvement reduced inappropriate outpatient antimicrobial therapy by 39% among a small number of patients requiring therapy modification [6]. Similarly, our study found an absolute improvement in appropriate antimicrobial therapy after implementation of a pharmacist-led TPAD service by 23.0 percentage points among all patients in the TPAD group and by 50.9 percentage points in the subset of patients requiring a prescription change. Van Abel et al likewise described postdischarge antimicrobial stewardship pharmacist review of finalized results, but their workflow differed in important ways from ours, including exclusion of urine cultures and inclusion requiring inpatient infectious diseases consultation, both of which likely selected for a different case mix [11]. Median time from culture update to pharmacist review in Van Abel et al was 27.2 hours, shorter than the median time to documentation observed in our study, likely due to our thrice-weekly review model. However, in our study documentation often occurred after clinician communication, pharmacy coordination, or delayed patient contact, so the documentation timestamp may not fully capture when action was initiated.

Our study also highlights the potential advantages of a facilitated stewardship model compared with passive notification alone. Prior automated alert studies, including work by El-Kareh et al and Dalal et al, improved or shortened follow-up in some respects but left substantial gaps in documented acknowledgment and action [7, 8]. Darragh et al have emphasized the complexity of this problem and asserted that multifaceted interventions are needed to address TPAD [2]. Whitehead et al's review of interventions addressing TPAD includes approaches focused on discharge summary education, electronic tools, notifications, and patient portals but interventions incorporating a clinician who actively interprets the result and facilitates next steps are notably absent [1, 2]. Our TPAD service combined result review with clinical interpretation, selective provider notification, facilitation of outpatient prescription changes, and patient counseling. Not all postdischarge infectious diseases results warrants action, and indiscriminate notification creates more risk for alert fatigue and may inadvertently promote overtreatment of findings such as asymptomatic bacteriuria, contamination, or colonization. Our model was intentionally designed to triage which results required clinician engagement and to first contact the provider most familiar with the acute hospitalization. Our findings support the added value of human facilitation in the TPAD workflow.

Although pharmacist review and documentation improved substantially after implementation of the TPAD service within our study, opportunities for improvement remain. The gap in pharmacist review reflected limitations in prospective case identification. Because the TheraDoc workflow required manual maintenance of unit mappings and adjustment of report look-back periods following pharmacist absences, eligible results could be missed when these parameters were not updated. The specific cause was not identifiable for every missed case, highlighting the value of automated, comprehensive case-finding systems with fewer manually maintained parameters. Documentation of TPAD acknowledgment or action in the EHR increased compared with preintervention performance and exceeded rates reported in prior reports but remained incomplete at 50% [16]. Future studies should target systems-level strategies to improve documentation, such as streamlined EHR workflows, structured fields, or automated dashboards that better capture pharmacist review and downstream follow-up actions.

This study has several strengths. It evaluated a pragmatic intervention embedded in routine antimicrobial stewardship workflow at a community hospital, enhancing its real-world relevance. Bias was minimized by using an infectious diseases-trained pharmacist who was not involved in delivering the intervention to assess antimicrobial appropriateness. In addition, the adjusted analyses accounted for clinically relevant covariates. The TPAD service intentionally focused on clinically meaningful gaps in therapy rather than broader stewardship interventions such as de-escalation or duration optimization after discharge. The outcome therefore reflected clinically acceptable antimicrobial therapy rather than fully optimized antimicrobial prescribing. Regimens were not considered inappropriate solely because they were broader in spectrum or longer in duration than necessary; consequently, the observed effect primarily represents improvement in patient-safety–focused prescribing deficiencies rather than comprehensive spectrum and duration Say have produced a conservative estimate of the intervention's broader stewardship impact. Several limitations should be considered. First, this was a single-center, retrospective, pre–post study. Second, few 30-day infection-related events occurred, and the study was underpowered to detect meaningful differences between groups; therefore, no conclusions regarding the intervention's effect on clinical outcomes can be drawn from these data. Third, outcome capture was limited to events identifiable within available health system records, so events occurring outside the system were missed. Fourth, we did not systematically track unsuccessful patient contact attempts, which may have affected intervention completion. Fifth, prescription claims data were not fully available for all patients, including those receiving medications through the Veterans Affairs system. Sixth, although outcome adjudication was performed by an infectious diseases-trained pharmacist who was independent of intervention delivery, the adjudicator was not blinded to study period, introducing the possibility of outcome-assessment bias.

Despite these limitations, our findings have implications for generalizability and implementation. Notably, this service was delivered using a thrice-weekly review model rather than daily review, suggesting that hospitals without dedicated full-time TPAD personnel may still be able to implement a meaningful intervention. This approach may be especially useful for inpatient antimicrobial stewardship programs seeking to expand transitions-of-care activities or for hospitals that can leverage pharmacy faculty and supervised student extenders. Future work should evaluate this model in larger and more diverse settings, assess its impact on patient-centered and utilization outcomes, and explore operational refinements such as automated dashboards or enhanced identification tools to improve case capture and efficiency. Additional work is also needed to determine whether expanding the service to include de-escalation or duration optimization after discharge would further improve antimicrobial stewardship outcomes while balancing patient inconvenience and cost.

In summary, implementation of a pharmacist-led service to address infectious diseases TPAD was associated with improved outpatient antimicrobial appropriateness at 7 days postdischarge and a faster transition to appropriate therapy among patients requiring prescription modification. This pragmatic, sustainable model may offer a scalable approach for community hospitals seeking to strengthen antimicrobial stewardship during care transitions.

## Supplementary Material

ofag585_Supplementary_Data

## Supplementary Data

Supplementary materials are available at *Open Forum Infectious Diseases* online. Consisting of data provided by the authors to benefit the reader, the posted materials are not copyedited and are the sole responsibility of the authors, so questions or comments should be addressed to the corresponding author.

## Notes

***Author contributions.*** E.W.C. contributed to conceptualization, methodology, resources, data curation, writing—original draft, writing—review & editing, supervision, project administration, and funding acquisition. M.S.L. contributed to methodology, software, validation, formal analysis, resources, writing—original draft, writing—review & editing, and visualization. J.P. contributed to methodology, software, validation, formal analysis, resources, writing—original draft, writing—review & editing, visualization, and funding acquisition. L.L. involved in data curation, writing—original draft, writing—review & editing, and project administration. M.B. contributed to data curation and writing—review & editing. S.G.G. involved in conceptualization, methodology, resources, data curation, writing—original draft, and writing—review & editing.

***Data availability***. The data that support the findings of this study are not publicly available due to privacy or institutional restrictions.

***Financial support.*** This work was supported by the Auburn University Research Support Program (RSP). The funder had no role in study design, data collection, analysis, or manuscript preparation.

***Potential conflicts of interest.*** All authors: No reported conflicts.

***Patient consent statement.*** This study was approved by the East Alabama Health Institutional Review Board (protocol 24-27-E) and the Auburn University Institutional Review Board (protocol STUDY00000310), both with a waiver of informed consent. The study did not include direct patient contact, and all procedures were conducted in accordance with applicable ethical standards.

***Use of artificial intelligence (AI) tools***. Artificial intelligence (AI) tools (M365 Copilot based on GPT-5) were used to assist with language editing, refinement of manuscript text, and preparation of supporting statements (eg, acknowledgments, funding, and formatting elements). All AI-generated content was reviewed, edited, and verified by the authors, who take full responsibility for the content of the manuscript.
