# Supplementary material for: Pharmacist-led Transitions of Care Intervention to Address Infectious Disease Tests Pending at Discharge (TPAD)
Source: Open Forum Infect Dis. 2026 Sep 12;13(9):ofag585. doi: 10.1093/ofid/ofag585 (PMC13590227; doi:10.1093/ofid/ofag585)
Supplement: Supplementary file 1 [file ofag585_supplementary_data.docx]

**Supplemental Table 1. TPAD Intervention Components Mapped to i-PARIHS Construct**

| **i-PARIHS construct** | **TPAD intervention mapping** |
| --- | --- |
| **Innovation** | Pharmacist-led inpatient culture follow-up service to identify post-discharge infectious disease results, assess antimicrobial appropriateness, recommend changes, facilitate prescription modification, and counsel patients. |
| **Recipients** | Stewardship/clinical pharmacist, prescribing providers, laboratory personnel, community pharmacy staff, and patients receiving post-discharge counseling. |
| **Context** | East Alabama Medical Center discharge workflow; use of Cerner and TheraDoc; transition from inpatient to outpatient care; operational barriers including patient identification, distinguishing inpatient vs observation status, identifying the responsible provider, and reaching patients after discharge. |
| **Facilitation** | The pharmacist served as the active facilitator by collaborating across disciplines to design the service, reviewing alerts, communicating recommendations to providers, supporting prescription changes, and counseling patients. |
